# Impact of intramammary inoculation of inactivated *Lactobacillus rhamnosus* and antibiotics on the milk microbiota of water buffalo with subclinical mastitis

**DOI:** 10.1371/journal.pone.0210204

**Published:** 2019-01-07

**Authors:** Carlotta Catozzi, Anna Cuscó, Cristina Lecchi, Esterina De Carlo, Domenico Vecchio, Alessandra Martucciello, Luisa D’Angelo, Olga Francino, Armand Sanchez Bonastre, Fabrizio Ceciliani

**Affiliations:** 1 Dipartimento di Medicina Veterinaria, Università di Milano, Milano, Italy; 2 Vetgenomics. Ed Eureka. PRUAB. Campus UAB, Barcelona, Spain; 3 Istituto Zooprofilattico Sperimentale del Mezzogiorno, National Reference Centre for Hygiene and Technologies of Water Buffalo Farming and Productions, Salerno, Italy; 4 Molecular Genetics Veterinary Service (SVGM), Veterinary School, Universitat Autònoma de Barcelona, Barcelona, Spain; Tokat Gaziosmanpasa University, TURKEY

## Abstract

Water buffalo mastitis represents a major issue in terms of animal health, cost of therapy, premature culling and decreased milk yeld. The emergence of antibiotic resistance has led to investigate strategies to avoid or reduce antibiotics’ based therapies, in particular during subclinical mastitis. The use of Generally Regarded As Safe bacteria (GRAS) such as *Lactobacillus rhamnosus* to restore the unbalance in mammary gland microbiota could provide potential corrective measures. The aim of this study was to investigate the changes in milk microbiota after the intramammary treatment with inactivated cultures of *Lactobacillus rhamnosus* of mammary gland quarters naturally affected by subclinical mastitis as compared to antibiotic therapy.A number of 43 quarters affected by subclinical mastitis with no signs of clinical inflammation and aerobic culture positive for pathogens were included in the study. The experimental design was as follows: 11 quarters were treated with antibiotics, 15 with inactivated cultures of *Lactobacillus rhmnosus* and 17 with PBS as negative control, by means of intrammary injection. Samples were collected at eight time points, pre- (T-29, T-21, T-15, T-7, T0 days) and post- treatment (T1, T2, and T6 days). Microbiological culture and Somatic Cell Count (SCC) were perfomed on all the samples, and microbiota was determined on milk samples collected at T0 and T6 by amplifying the V4 region of 16S rRNA gene by PCR and sequencing using next generation sequencing technique. Treatment with *Lactobacillus rhamnosus* elicited a strong chemotactic response, as determined by a significant increase of leukocytes in milk, but did not change the microbiological culture results of the treated quarters. For what concerns the analysis of the microbiota, the treatment with *Lactobacillus rhamnosus* induced the modification in relative abundance of some genera such as *Pseudomonas* and *5-7N15*. As expected, antibiotic treatment caused major changes in microbiota structure with an increase of *Methylobacterium* relative abundance. No changes were detected after PBS treatment. In conclusion, the present findings demonstrated that the *in vivo* intrammmary treatment with *Lactobacillus rhamnosus* has a transient pro-inflammatory activity by increasing SCC and is capable to modify the microbiota of milk after six days from inoculation, albeit slightly, even when the bacterial cultures were heat inactivated. Further studies are necessary to assess the potential use of this GRAS as supportive therapy against mastitis.

## Introduction

The domestic water buffalo (*Bubalus bubalis*) contributes to a significant share of global milk production and is the major milk producing animal in several countries, such as India and Pakistan [1]. Water buffaloes are resistant to most of the disease affecting dairy cows, even in a context of low feeding and environmental stress [2–4]. The background of this resistance lies in mammary gland anatomical features, including a long narrow teat canal, a teat skin less sensitive to chapping and sores, a streak canal with thicker epithelium and keratin layer, a tighter sphincter of streak canal and the absence of milk cistern [5]. These distinct features of the buffalo mammary gland are believed to prevent the invasion of micro-organisms. The few studies on water buffalo mastitis presented the evidence that somatic cell score in quarters with intramammary infection is low, and a limited decrease in milk production was found among infected animals as compared to healthy ones [6]. However, mastitis is still occurring in dairy buffaloes in intensive dairy farming [7], with an impact that might be comparable to that of dairy cows concerning production losses, culling and treatment costs [8], beside decreasing animal health and welfare [9,10].

The conventional therapy against mastitis includes the treatment of the mammary gland with antibiotics. Although necessary for both therapeutic and prophylactic purposes, treatment with antibiotics is not fully efficient, and presents several drawbacks. The extended use of antibiotics is at the background of the development of anti-microbial resistance that can persist in the bacterial community [11, 12], as demonstrated for *Streptococcus agalactiae* [13] and *Staphylococcus aureus* [14]. Furthermore, the massive use of antibiotics in dairy animals is at the origin of antibiotic residues’ pollution in the environment and contamination of milk and other animal-derived products, causing antibiotic resistance in humans as well [15].

Alternative strategies are investigated, aiming to reduce the use of antibiotics. New therapeutic approaches, such as, among the others, Generally Recognized As Safe (GRAS) bacteria, including Lactic Acid Bacteria (LAB), have been developed [16]. The *in vitro* and *in vivo* effects of treatment with LAB produced different and opposite results in cows. *In vitro* studies on the effects of *Lactococcus lactis* as potential anti-mastitis therapeutics have shown promising results on bovine mammary epithelial cells by producing nisin A, a polycyclic antibacterial peptide [17]. The different strains of lactobacilli that have been investigated so far included *Lactobacillus perolens*, *Lactobacllus rhamnosus*, *Lactobacillus brevis* and *Lactobacillus plantarum* [18–21]. *In vitro* results were encouraging, and an overall reduction of bacterial load together with an anti-inflammatory activity were demonstrated. On the contrary, the *in vivo* use of GRAS produced contradictory results and their activity remains inconclusive. *Lactococcus lactis* stimulates the intramammary immune system of cattle, as determined by polymorphonuclear cells (PMN) recruitment and increasing of haptoglobin and serum amyloid A concentrations in milk [22]. Nonetheless, only in few studies the live cultures of *Lactococcus lactis* were effective in bovine mastitis treatment [23]. In a model of mouse mastitis, the experimental infection with *Staphylococcus chromogenes* and treatment with live cultures of *Lactococcus lactis* induced an increased level of IL-1β and TNFα, in addition to tissues damages, suggesting that these GRAS strains cannot be used for mastitis treatment in rodents. Recent findings on ewes affected by subclinical mastitis confirmed that the infusion of *Lactococcus lactis* into the mammary gland leads to a transient clearance of the pathogens, but also increases the inflammatory status of the mammary gland [24]. Similarly, treatment with different strains of *Lactobacillus* failed to decrease cow mastitis and caused a local inflammatory response [25,26]. Among GRAS, *Lactobacillus rhamnosus* was found to possess the strongest antibacterial activity against *Salmonella enterica* [27], and also capable of preventing the *Escherichia coli*–induced changes in epithelial barrier functions [28]. Similar results were demonstrated in cows as well, where the potential of *Lactobacillus rhamnosus* against *Escherichia coli*-infection in vagina and endometrium [29,30], intestine [31] and respiratory apparatus [32] was also reported. Information about the activity of *Lactobacillus rhamnosus* on mammary gland is, one the contrary, very limited. *In vitro* studies provided evidence that *Lactobacilus rhamnosus* pretreatment was able to attenuate the pro-inflammatory effects of an *E*.*coli* challenge on primary bovine mammary epithelial cells by suppressing TLR and inflammasome related gene expression [33,34]. To the best of the knowledge of the authors, no study was carried out to investigate the *in vivo* effects of *Lactobacillus rhamnosus* in the mammary gland, in particular for what concerns how *Lactobacillus* treatment can influence the delicate equilibrium between bacterial communities.

Culture-independent techniques relying on high-throughput DNA sequencing of 16S provided an in-depth knowledge of bacterial communities, and are currently applied to unravel the relationship between resident microbial population and the development of mastitis[35–38]. The results of these studies demonstrated that bacterial species are present in culture-negative samples collected from animals with clinical mastitis [39] and that major pathogens, such as *Streptococcus uberis* and *Staphylococcus aureus*, can be found in milk from clinically healthy animals [35]. On this background, the insurgency of mastitis may be related to both the presence of specific pathogen and the modification of the microbial community of milk [40]. This observation was confirmed in water buffalo, whose milk microbiota has been recently published [41].

The aim of this study was to investigate the effect of an intramammary inoculation of *Lactobacillus rhamnosus* on the milk SCC and microbiota of water buffaloes naturally affected by sub-clinical mastitis. The effect of antibiotics, that were also used as positive control for anti-bacterial activity on milk microbiota, were characterized as well. *Lactobacillus rhamnosus* was selected on the background of its *in vitro* antibacterial activity in the epithelial mammary gland cellular model.

## Materials and methods

### Bacterial strain, culture conditions and inactivation of *Lactobacillus rhamnosus* inocula

The probiotic *Lactobacillus rhamnosus* strain GG (LMG 18243) from the BCCM/LMG Bacteria Collection (Belgium) was prepared as follows: the bacterium was grown at 37°C for 48h in Trypticase Soy Broth (TSB, BD, Italy) in a Gaspak jar using the commercial gas-generating AnaeroGen AN25 kit (Oxoid, England) for anaerobic growth. The probiotic culture was then centrifuged at 3000 x g for 20 min, washed twice with sterile pyrogen-free saline solution (NaCl 0.9%) and suspended in the solution used for the inoculum, namely sterile PBS (Sigma-Aldrich, Milano). This bacterial suspension (approximately 10^9^ CFU x mL^-1^) was inactivated after boiling at 100°C for 15 min. The absence of viable cells was verified by culturing on TBS medium. Five mL of the heat-inactivated suspension were used for each intramammary injection.

### Study design and intramammary challenge

The experimental protocol was approved by Italian Ministry of Health (Protocol No.982/2015PR). The study was carried out on 20 multiparous water buffaloes (*Bubalus bubalis*) homogeneous for parity (2^nd^ to 4^th^ lactation) and in mid lactation (from 60 to 160 DIM). The animals were housed in a commercial farm and left 29 days to become familiar with the experimental conditions. During that time, animal health status was diagnosed clinically and quarter milk samples were collected for bacteriological analysis and Somatic Cell Count (SCC).

For the purpose of this study, quarters affected by sub-clinical mastitis were defined as those with no evidence of clinical signs, but positive to microbiological culture for three times before T0 (included). Following these criteria, a total number of 43 samples were included in the study as affected by sub-clinical mastitis. Milk samples were collected weekly at T-29, T-21, T-15, T-7 and T0 and then intramammary inoculated following this protocol: at T0, 15 quarters were inoculated with 5 ml of inactivated cultures of *Lactobacillus rhamnosus* (LAB) (LAB-T0), 11 quarters were inoculated with amoxicillin-clavulanic acid (Synulox Lactating Cow Intramammary Suspension, Pfizer, Italy) (Ab-T0), and 17 quarters were inoculated with 5 ml of sterile PBS (Sigma-Aldrich, Milano) (PBS-T0). After challenging, samples of milk were further collected at time T1, T2 and T6.

Milk samples were collected after disinfection of teat ends with a 2% povidone-iodine (Betadine Solution) and discarding of the first three strains of milk. Gloves were changed each time and 150 ml of milk were collected in sterile containers. After collection, milk samples were immediately refrigerated and delivered to the laboratory for microbiological analysis and SCC. Milk samples were finally aliquoted and stored at -80 C for microbiota identification, which was carried out on milk samples at T0 and T6.

### Clinical observation and animal care

Clinical signs were monitored throughout the experiment by a veterinary practitioner, every 8 hours during the first 24 hours from the challenge and then every time the water buffaloes were milked. Rectal temperature was measured every 24 hours. General attitude, and appetite were evaluated, and the udders were palpated to identify soreness, swelling hardness and heat, to assess the development of clinical signs.

### Microbiological culture (MC) and Somatic Cell Count (SCC)

Microbiological culture tests were performed for each milk sample using different media as previously reported [41]. Briefly, samples were incubated at 37° for 24h in aerobic conditions on Trypticase soy agar (with 5% sheep blood), MacConkey agar and Baird Parker agar; at 37° for 72h in aerobic conditions on Prototheca isolation medium (PIM); at 37° in microaerobic conditions on Mycoplasma agar. Gram staining, coagulase and oxidase tests were performed on cultures with mastitis pathogens; in particular, in *Staphylococcus* spp. positive culture were tested for coagulase activity using rabbit plasma, and *Streptococcus* spp positive cultures were evaluated with Streptokit-BioMérieux test for Lancefield grouping. Somatic cell count was measured in milk samples at T-29, T-21, T-15, T-7, T0, T1, T2, and T6 (days) using Fossomatic (Foss) apparatus by means of the UNI EN ISO 13366–2:2007 technique for electronic optical fluorimetric counters.

### DNA extraction

One ml of milk was centrifuged at room temperature at 16,100 rcf [36,41]. Fat and supernatant were discarded and the remaining pellet was resuspended with 250ul of the Power Bead Tube of the DNEasy Power Soil Kit (QIAGEN) used to extract bacterial DNA, according to the manufacturer’s instructions. After the DNA elution in 50 μl of DNAse and RNAse free water, DNA concentration and purity were analysed using NanoDrop 2000 Spectrophotometer (Thermo Fisher Scientific, Weltham, Massachusetts, U.S.A.) at wavelength 230, 260 and 280 nm and DNA samples were stored at -80° until further processing. The reagents included in the kit, without any bacterial DNA, were used as blank control for each DNA extraction batch.

### Amplification of the hypervariable V4 region of bacterial 16S rRNA gene by PCR and barcoding

V4 region of 16S rRNA gene was amplified for each sample [37]. The forward primer was 5’–CCATCTCATCCCTGCGTGTCTCCGAC**TCAG***NNNNNNNNNNNNNNNNN***GAT**GTGYCAGCMGCCGCGGTAA– 3’, composed of the adapter linker, the key, the barcode, different for each sample, and the forward primer 515F. The reverse primer was 5’–CCTCTCTATGGGCAGTCGGTGATGGACTACNVGGGTWTCTAAT– 3’, composed of the adapter linker and the R806 reverse primer. The Thermo Scientific Phusion Hot Start II High-Fidelity DNA polymerase kit was used to perform V4 PCR; each PCR reaction contained RNAse and DNAse free water, 5x Phusion Buffer HF (5 μl), dNTPs 2mM (2.5 μl), Primer Fw 10μM (1.25 μl), Primer Rv 10μM (1.75 μl), Phusion High Fidelity Taq Polymerase 2 U/μl (0.25 μl) and 5 ng of DNA. When DNA samples quantification was too low (less than 5 ng/μl), 5 μl of the samples were used to perform PCR. The thermal profile consisted of an initial denaturation of 30 sec at 98°C, followed by 32 cycles of 15 sec at °98 C, 15 sec at 50°C, 20 sec at 72°C, and a final extension of 7 min at 72°C. Each PCR plate included samples derived from each group. After DNA purification using Agencourt AMPure XP kit with a ratio 1:1, quality and quantity of PCR products were determined using Agilent Bioanalyser 2100 and Qubit fluorometer.

For 17 samples showing DNA concentration lower than 1 ng/μl at Qubit quantification, PCR was repeated using the same PCR condition and increasing the number of cycles up to 36. The lack of amplification of extraction and PCR negative controls was confirmed for all PCR.

### Next-generation sequencing, bioinformatics and statistical analysis

Sequencing was performed using Ion Torrent Personal Genome Machine (PGM) with the Ion 318 Chip Kit v2 (Thermo Fisher Scientific, Weltham, Massachusetts, U.S.A.), by the Centre for Research in Agricultural Genomics (CRAG, Bellaterra, Barcelona), following manufacturer’s instructions. The raw sequences have been submitted to NCBI under Bioproject accession number SUB4205063—Bioproject number: PRJNA477950. After sequencing, reads were demultiplexed in order to have sequence file for each barcode/sample and Primer Rv was removed. Then, sequences were imported in the Quantitative Insight Into Microbial Ecology 2 (QIIME 2) software [42] (https://qiime2.org), which was used to analyze data. After obtaining a unique file with all sequencing data, DADA2 was used as quality filtering method in order to denoise, dereplicate single-end sequences and remove chimeras [43]. Afterward, the primer Fw was removed and a truncation length of 245 bases was used, taking into account the quality plot result and the mean V4 length of around 250 bases. After that, the units of observation, composed of unique sequences namely Amplicon Sequence Varians (ASVs), were used to classify them and assign taxonomy, using Greengenes 13.8 [44] at 99% of Operational Taxonomic Units (OTUs) identity and trimmed to V4 region, as reference database. Finally, chloroplasts were removed from the sequences.

The filtered feature table was used to perform the downstream analysis. The taxonomic analysis was performed for each sample or group of samples at phylum, family and genus level. Diversity analysis was assessed using 9500 sequences per sample. Alpha diversity that analyses differences within samples was performed using qualitative and quantitative approaches (richness or Observed species and evenness or Shannon index, respectively); beta diversity that analyzes differences among samples estimating how many taxa are shared among samples, was performed using qualitative and quantitative approaches as well (unweighted and weighted UniFrac distances matrices, respectively).

As data presented in this study were not-normally distributed and composed of pre- and post-treatment samples (T0 vs T6 within the same group), non-parametric paired test was applied. To compare the effect of the treatment on the microbiota (PBS-T6 vs LAB-T6; PBS-T6 vs Ab-T6; LAB-T6 vs Ab-T6), non-parametric unpaired test was applied. Taxonomic statistical analysis was performed using Wilcoxon signed pairwise test (pairwise.wilcox.test in coin package) and Kruskal Wallis test followed by Dunn pairwise test (dunn.test package) in R version 3.4.3 (http://www.R-project.org), for paired and unpaired comparisons, respectively. A specific QIIME 2 plugin for longitudinal studies was used for alpha diversity and beta diversity principal coordinates analyses: as two time points were considered for this experiment, Wilcoxon rank sum pairwise test was used for paired data, while Kruskal Wallis and Wilcoxon Mann-Whitney U pairwise test were applied for unpaired data [45]. Workflow details are available at dx.doi.org/10.17504/protocols.io.ucpesvn.

## Results

### Diagnosis of sub-clinical mastitis, intramammary inoculation of inactivated *Lactobacillus rhamnosus*, collection of samples

The diagnosis of sub-clinical mastitis was carried out according to microbiological culture results and SCC. None of the animals included in this study evidenced any clinical signs related to the development of an acute mastitis. Results of microbiological culture are presented in Tables 1 and S1 showing that, at T6, bacteria associated with mastitis were found in all the samples included in the study, except those collected from quarters treated with antibiotics, all of which became negative at microbiological culture at T6, with only one exception.

**Table 1:** Microbiological culture results for each treatment group.

	T0	T6
**PBS treated**	**17**	**17**
Staphylococcus aureus	**9**	**7**
Coagulase-negative Staphylococci	**4**	**3**
Streptococcus agalactiae	**1**	**0**
Staphylococcus aureus / Streptococcus agalactiae	**2**	**3**
Coagulase-negative Staphylococci / Streptococcus agalactiae	**1**	**1**
Negative	**0**	**3**
**LAB treated**	**15**	**15**
Staphylococcus aureus	**9**	**9**
Coagulase-negative Staphylococci	**3**	**2**
Streptococcus agalactiae	**3**	**0**
Staphylococcus aureus / Streptococcus agalactiae	**0**	**2**
Coagulase-negative Staphylococci / Streptococcus agalactiae	**0**	**2**
Negative	**0**	**0**
**Ab treated**	**11**	**11**
Staphylococcus aureus	**8**	**1**
Coagulase-negative Staphylococci	**2**	**0**
Streptococcus agalactiae	**0**	**0**
Staphylococcus aureus / Streptococcus agalactiae	**0**	**0**
Coagulase-negative Staphylococci / Streptococcus agalactiae	**1**	**0**
Negative	**0**	**10**

PBS: quarters treated with sterile PBS only, LAB: quarters treated with inactivated culture of *Lactobacillus rhamnosus* only, Ab: quarters treated with antibiotics, as described in Material and Methods. T0: pre-treatment time; T6: time at 6 days post treatment.

**Fig 1:**
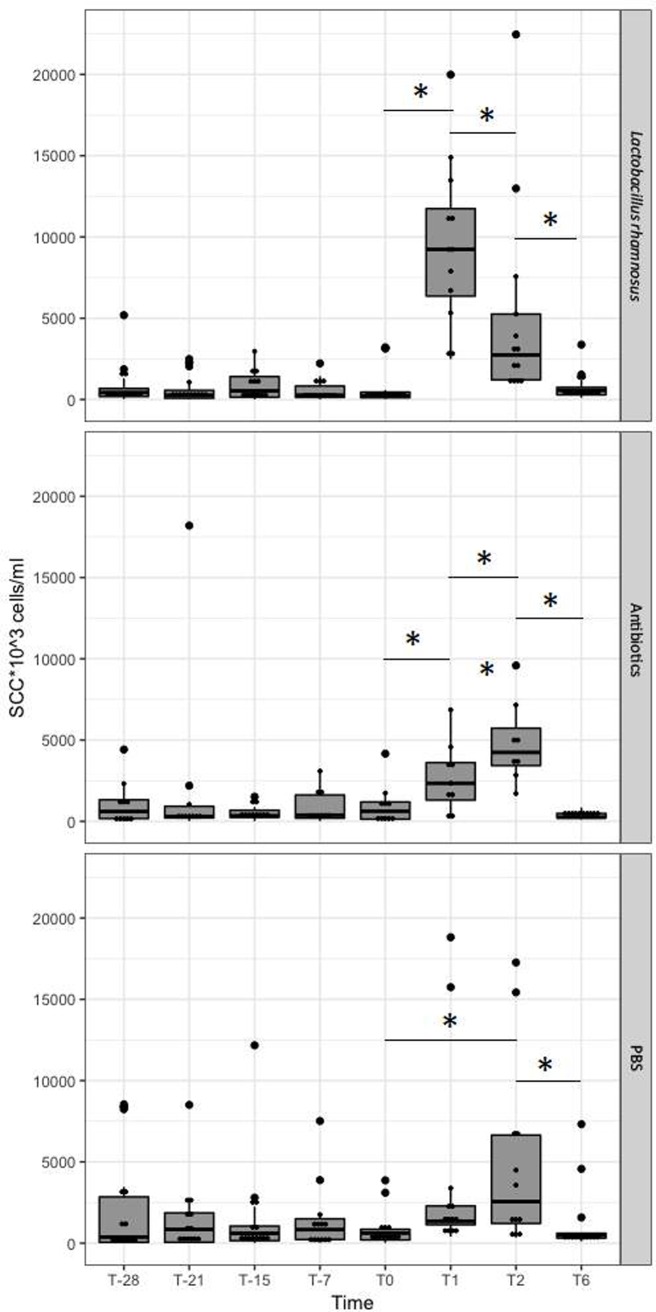
Somatic cell count in response to challenge with LAB, Ab and PBS. Median (line into the box), upper and lower quartiles (ends of the box) and highest and lowest values (extreme lines) are shown for challenged quarters. The bigger black points indicate outliers. * indicates statistical significance at *p < 0*.*05*.

Somatic Cell Counts were measured at T-29, T-21, T-15, T-7 and T0, with the aim to monitor the microbial status of each quarter and identify those that would be included in the study, and T1, T2, and T6, to assess the effects of the treatment. All quarters challenged with inactivated *Lactobacillus rhamnosus* showed an increase in SCC. In individual quarters, elevation of SCC median reached its peak 24h post inoculation and then decreased afterward (Fig 1). PBS-infused control quarters showed a significant increase in SCC as compared with prechallenge levels starting from T1, and increased after T2. Antibiotic treated quarters showed an increase in SCC starting from T1 and further increases at T2. At T6, the SCC were decreased at the T0 level in all the three groups of samples.

### Ion Torrent output: Sequences results after filtering procedures

The sequencing of 43 samples produced a total of 9,468,300 sequences and 4,039 features were obtained (with a mean of 112,717.85, a minimum of 9,778 and a maximum of 500,775 sequences) after filtering.

### Core microbiota and taxonomic profile analysis before and after the treatment

**Fig 2:**
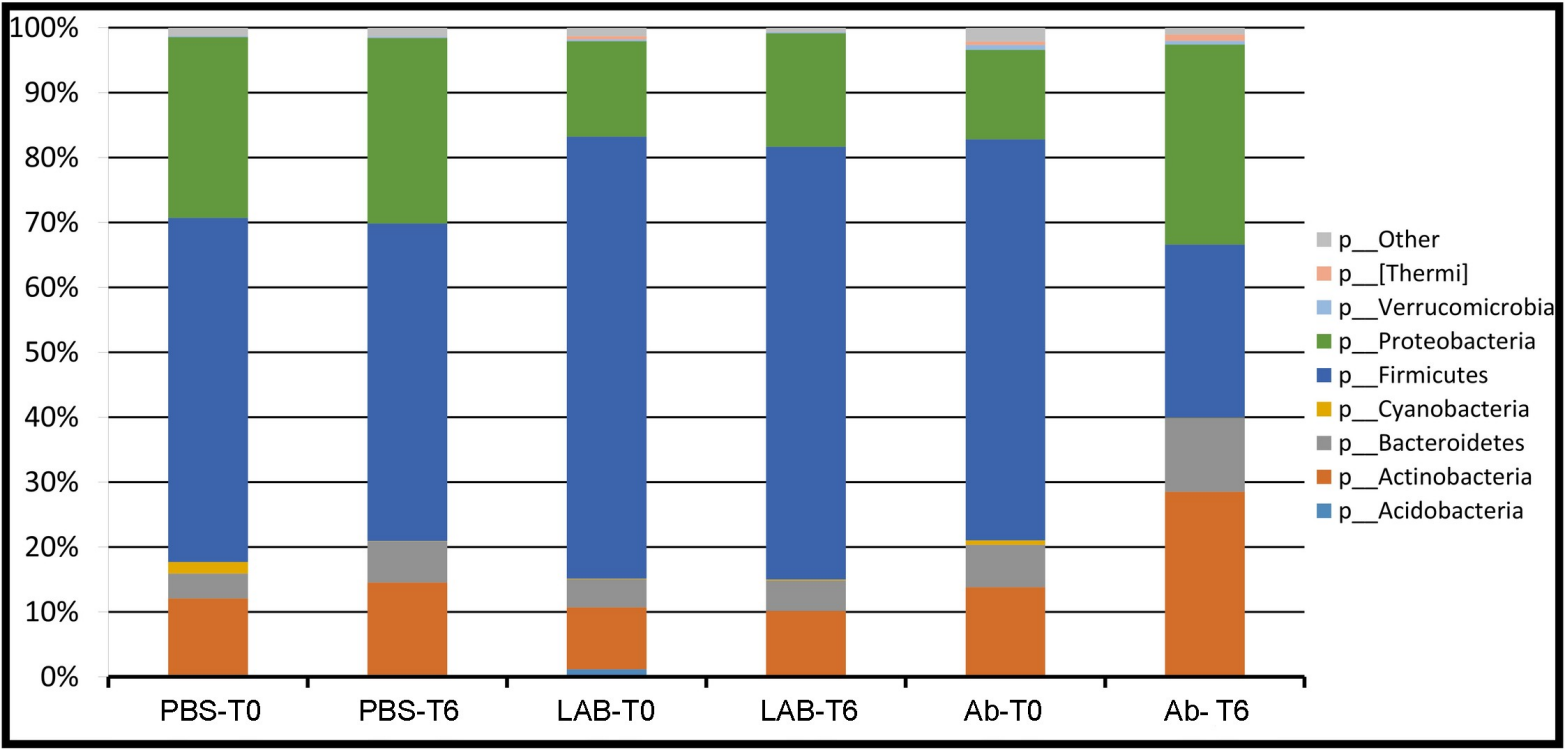
Water buffalo milk taxonomic profile at phylum level. LAB: quarters treated with inactivated culture of *Lactobacillus rhamnosus* only, Ab: quarters treated with antibiotics, PBS: quarters treated with sterile PBS only. T0: time zero; T6: time at 6 days post treatment.

The core microbiota of milk from water buffaloes affected by subclinical mastitis is composed of eight main phyla, namely *Acidobacteria*, *Actinobacteria*, *Bacteroidetes*, *Cyanobacteria*, *Firmicutes*, *Proteobacteria*, *Verrucomicrobia* and *[Thermi]*. Results are presented in Fig 2 and Table 2. The milk microbiota was dominated by Firmicutes (mean of 60.9% at T0) and Proteobacteria (mean of 18.8% at T0). Treatment with *Lactobacillus rhamnosus* and PBS did not induce any major change from T0 to T6, with the exception of *[Thermi]*. On the contrary, treatment with Ab induced a decrease of Firmicutes (from 61.8% at T0 to 26.7% at T6), and an increase of Proteobacteria (from 13.8% at T0 to 30.8% at T6) and Actinobacteria (from 13.8% at T0 to 28.5% at T6). Comparing the relative abundance of bacterial phyla at T6 between different treatments, no differences were found between treatment with PBS and treatment with *Lactobacillus rhamnosus*. On the contrary, several differences were found between milk microbiota from quarters treated with *Lactobacillus rhamnosus* and Ab in the relative abundance of *Acidobacteria*, *Cyanobacteria and Firmicutes*. Differences were also found between milk quarters treated with PBS and Ab, in the relative abundance of *Actinobacteria* and *Firmicutes*.

**Table 2:** Relative abundance of microbiota taxa at phylum level.

	*Relative abundance frequencies*											
	Quarter treated with PBS		Quarters treated with LAB		Quarters treated with antibiotics		PBS	LAB	Ab	T6		
	PBS-T0	PBS-T6	LAB-T0	LAB-T6	Ab-T0	Ab-T6	T0 vs T6	T0 vs T6	T0 vs T6	PBS vs LAB	PBS vs Ab	LAB vs Ab
*Acidobacteria*	0,3%	0,3%	1,2%	0,2%	0,0%	0,0%	ns	ns	ns	ns	ns	ns
*Actinobacteria*	11,7%	14,2%	9,5%	9,9%	13,8%	28,5%	ns	ns	0,01	ns	0,002	<0,001
*Bacteroidetes*	3,9%	6,3%	4,3%	4,7%	6,5%	11,4%	ns	ns	ns	ns	ns	ns
*Cyanobacteria*	1,8%	0,0%	0,1%	0,1%	0,7%	0,0%	ns	ns	ns	ns	ns	0,01
*Firmicutes*	53,0%	48,9%	68,1%	66,7%	61,8%	26,7%	ns	ns	0,005	ns	0,01	0.01
*Proteobacteria*	27,9%	28,6%	14,7%	17,5%	13,8%	30,8%	ns	ns	0,04	ns	ns	ns
*Verrucomicrobia*	0,3%	0,2%	0,3%	0,2%	0,7%	0,5%	ns	ns	ns	ns	ns	ns
*[Thermi]*	0,0%	0,0%	0,5%	0,0%	0,6%	1,0%	ns	0,03	ns	ns	ns	ns
*Other*	1,1%	1.5%	1,3%	0,6%	2,1%	1,0%	ns	ns	ns	ns	ns	ns

LAB: quarters treated with inactivated culture of *Lactobacillus rhamnosus* only, Ab: quarters treated with antibiotics, PBS: quarters treated with sterile PBS only. T0: time zero; T6: time at 6 days post treatment

No core microbiota was present at family level. The main families found in milk microbiota at T0 were *Staphylococcaceae* (mean of 40.3%), followed by *Streptococcaceae* (mean of 5.8%), *Moraxellaceae* (mean of 5.2%), *Ruminococcaceae* (mean of 3.2%) and *Corynebacteriaceae* (mean of 4.4%). Taxonomic and statistical results at family level are shown in S1 Table and S1 Fig (relative abundance of almost 1%). PBS treatment did not cause significant milk microbiota alterations except for *Enterobacteriaceae* and *Rhodobacteriaceae*. Similarly, *Lactobacillus rhamnosus* treatment induced only an increase of *Pseudomonadaceae* (from 1.5% at T0 to 5.1% at T6). The main changes were present in antibiotic group at T6, where an increase of *Micrococcaceae* (from 0.9% at T0 to 3% at T6), *Bradyrhizobiaceae* (from 0.2% at T0 to 1.4% at T6), *Methylobacteriaceae* (from 1.1% at T0 to 6.7% at T6) and *Rhodocyclaceae* (from 0.1% at T0 to 1.2% at T6) was observed. A decrease of *Staphylococcaceae* (from 42.5% at T0 to 7.5% at T6) was also observed, even if the difference was not statistically significant. Comparing the relative abundance of taxa at family level among groups at T6, no differences were found between Ab and PBS groups except for *Microbacteriaceae* and *Cytophagaceae* and no major changes between *Lactobacillus rhamnosus* and PBS groups, except for *Peptostreptococcaceae* and *Comamonadaceae*. On the contrary, several changes were present between *Lactobacillus rhamnosus* and Ab microbiota at T6 showing differences in relative abundance (RA) of *Micrococcaceae*, *Propionibacteriaceae*, *Staphylococcaceae*, *Peptostreptococcaceae*, and *Comamonadaceae*.

**Fig 3:**
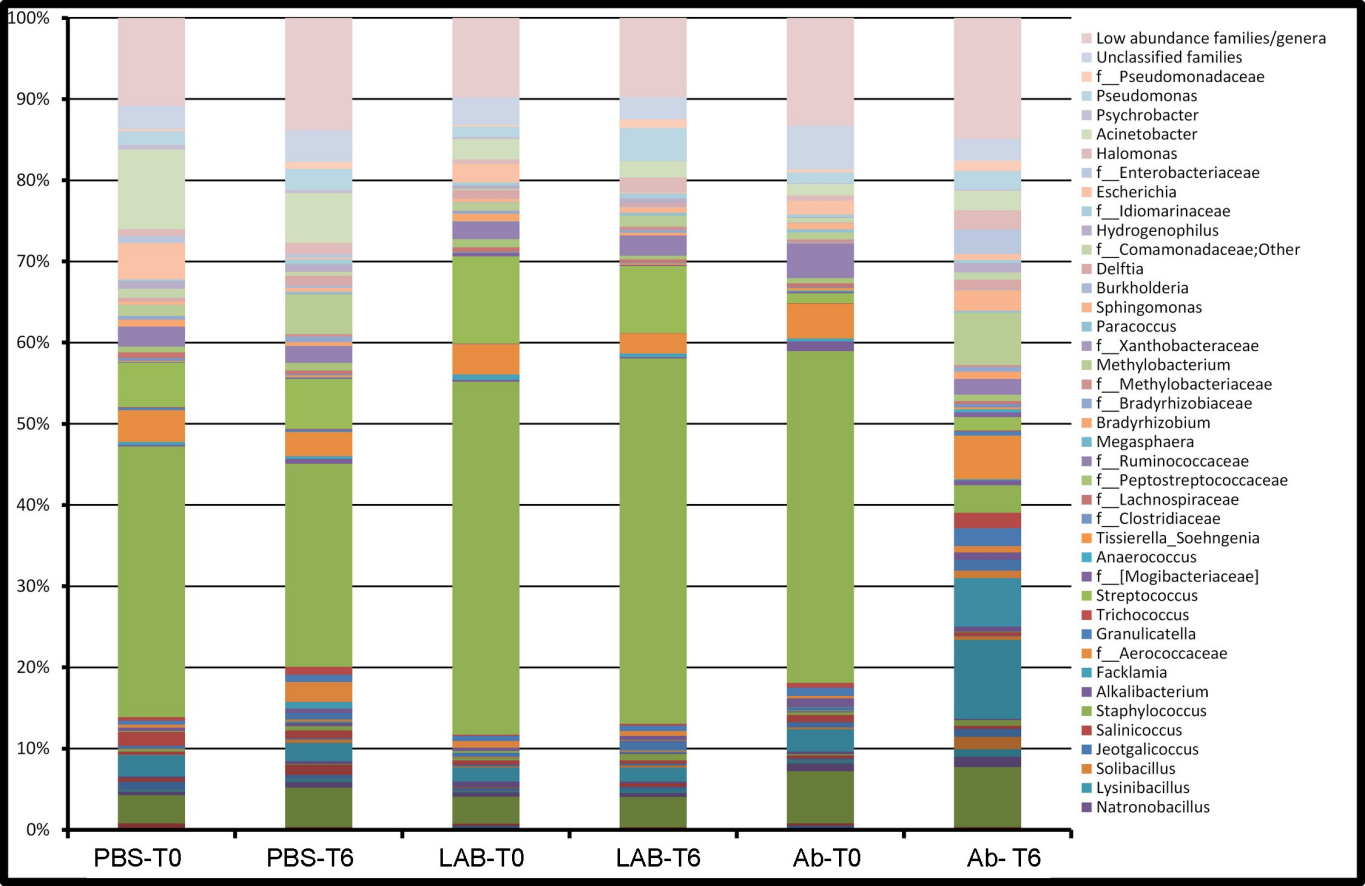
Water buffalo milk taxonomic profile at family/genus level (relative abundance of almost 1%). LAB: quarters treated with inactivated culture of *Lactobacillus rhamnosus* only, Ab: quarters treated with antibiotics, PBS: quarters treated with sterile PBS only, as described in Material and Methods. T0: time zero; T6: time at 6 days post treatment.

It was not possible to identify any core microbiota at genus level. Taxonomic and statistical results at genus level were shown in Table 3 and Fig 3 (relative abundance of almost 1%). Family level was indicated where genus level could not be reached. Milk samples at T0 were dominated by *Staphylococcus* (mean of 40%), followed by *Streptococcus* (mean of 9%), *Acinetobacter* (mean of 4.6%), *Corynebacterium* (mean of 4%) and *Propionibacterium* (mean of 2.3%). No changes were detected after PBS treatment. The main statistically significant changes after *Lactobacillus rhamnosus* treatment were identified as an increase of the RA of *Pseudomonas* from 1% at T0 to 4% at T6 and a minor increase of 5-7N15. As expected, major changes were found after Ab treatment, which induced a decrease of *Staphylococcus* from 41% at T0 to 3% at T6. A statistically significant increase of *Methylobacterium* was also found (from 1% at T0 to 6% at T6). Comparing the relative abundance of taxa at genus level among groups at T6, negligible changes were detected between PBS and *Lactobacillus rhamnosus* groups as well as between PBS and Ab groups. More genera differed between *Lactobacillus rhamnosus* and Ab microbiota, namely *Staphylococcus*, *Propionibacterium* and *5-7N15*.

**Table 3:** Relative abundance of microbiota taxa at family/genus level.

	*Relative abundance frequencies*						*p-value (where p < 0*.*05)*					
	Quarter treated with PBS		Quarters treated with LAB		Quarters treated with antibiotics		PBS	LAB	Ab	T6		
	PBS-T0	PBS-T6	LAB-T0	LAB-T6	Ab-T0	Ab-T6	T0 vs T6	T0 vs T6	T0 vs T6	PBS vs LAB	PBS vs Ab	LAB vs Ab
**Deinococcus**	0%	0%	0%	0%	1%	0%	ns	ns	ns	ns	ns	ns
**Corynebacterium**	3%	5%	3%	4%	6%	7%	ns	ns	ns	ns	ns	0,04
**Dietzia**	0%	1%	1%	0%	1%	1%	ns	ns	ns	ns	ns	ns
**Nesterenkonia**	1%	1%	0%	0%	0%	1%	ns	ns	ns	ns	ns	ns
**Rhodococcus**	0%	0%	0%	0%	0%	1%	ns	ns	ns	ns	ns	ns
**Propionibacterium**	3%	2%	2%	2%	3%	10%	ns	ns	ns	ns	ns	0,002
**CF231**	0%	0%	0%	0%	1%	0%	ns	ns	0.03	ns	ns	ns
**5-7N15**	0%	1%	0%	1%	0%	0%	ns	0.01	ns	ns	ns	ns
**Hymenobacter**	0%	0%	0%	0%	0%	6%	ns	ns	ns	ns	ns	ns
**Chryseobacterium**	0%	0%	0%	0%	0%	1%	ns	ns	ns	ns	0,04	ns
**Natronobacillus**	0%	1%	0%	1%	1%	1%	ns	ns	ns	ns	ns	ns
**Lysinibacillus**	0%	1%	0%	0%	0%	0%	ns	ns	ns	ns	ns	ns
**Solibacillus**	0%	2%	1%	1%	0%	1%	ns	ns	ns	ns	ns	ns
**Jeotgalicoccus**	0%	1%	1%	1%	1%	2%	ns	ns	ns	ns	ns	ns
**Salinicoccus**	0%	1%	0%	0%	1%	2%	ns	ns	ns	ns	ns	ns
**Staphylococcus**	33%	25%	43%	45%	41%	3%	ns	ns	0.03	ns	ns	0,01
**Alkalibacterium**	0%	1%	0%	0%	1%	1%	ns	ns	ns	ns	ns	ns
**Facklamia**	0%	0%	1%	0%	0%	0%	ns	ns	ns	ns	ns	ns
**Granulicatella**	0%	0%	0%	0%	0%	1%	ns	ns	ns	ns	ns	ns
**Streptococcus**	6%	6%	11%	8%	1%	2%	ns	ns	ns	ns	ns	ns
**Bradyrhizobium**	1%	0%	1%	0%	0%	1%	ns	ns	ns	ns	ns	ns
**Methylobacterium**	1%	5%	1%	1%	1%	6%	ns	ns	0,02	ns	ns	ns
**Sphingomonas**	0%	0%	0%	1%	1%	3%	ns	ns	ns	ns	ns	ns
**Delftia**	0%	1%	1%	0%	0%	1%	ns	ns	ns	ns	ns	ns
**Hydrogenophilus**	1%	1%	0%	1%	0%	1%	ns	ns	ns	ns	ns	ns
**Escherichia**	4%	0%	2%	0%	2%	1%	ns	ns	ns	ns	ns	ns
**Halomonas**	1%	1%	0%	2%	1%	2%	ns	ns	ns	ns	ns	ns
**Acinetobacter**	10%	6%	3%	2%	1%	2%	ns	ns	ns	ns	ns	ns
**Psychrobacter**	1%	0%	0%	0%	0%	0%	ns	ns	ns	ns	ns	ns
**Pseudomonas**	2%	3%	1%	4%	1%	2%	ns	0,007	ns	ns	ns	ns

LAB: quarters treated with inactivated culture of Lactobacillus rhamnosus only, Ab: quarters treated with antibiotics, PBS: quarters treated with sterile PBS only, as described in Material and Methods. T0: time zero; T6: time at 6 days post treatment

### Discriminant analysis following treatment

Considering the effect of the treatment on microbiota, alpha diversity showed differences between Ab- and LAB-treated groups at richness level, where a decrease of 85.4 and an increase of 80.3 observed species was observed, respectively (*p* = 0.03). No modification of richness or evenness was observed comparing microbiota T0 vs T6 within the same group.

**Fig 4:**
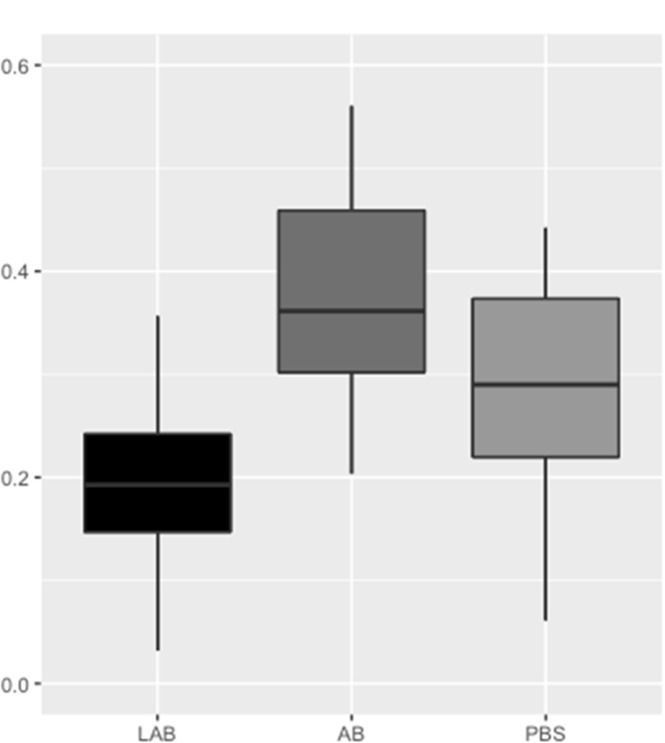
Boxplots show quartile distribution of weighted UniFrac distances between each group diversity after LAB, Ab and PBS treatment. Statistical significant differences were found between Ab and LAB (*p* = 0.001) and LAB and PBS (*p* = 0.003). LAB: quarters treated with inactivated culture of *Lactobacillus rhamnosus* only, Ab: quarters treated with antibiotics, PBS: quarters treated with sterile PBS only, as described in Material and Methods.

Beta diversity analysis showed differences on the basis of the weighted UniFrac distance matrix. Modification in microbiota was observed only after Ab treatment, whose groups at T0 and T6 were discriminated by the axis 2 from PCoA plot (*p* = 0.04): samples moved across the axis 2 in the same direction between T0 and T6, suggesting that these samples experienced the same directional shift in terms of microbiota structure, even if the magnitude or the final composition could not be the same. The effect of the treatment, plotted in Fig 4, showed that the Ab effect on the microbiota structure was greater than the *Lactobacillus rhamnosus* effect (*p* = 0.001), which was in turn smaller than the PBS effect (*p* = 0.003).

## Discussion

Probiotics have been used as a corrective measure to re-equilibrate the microbiota during mastitis, with contradictory results. Remarkably, the effects of GRAS on microbiota as determined by culture independent methods has not been investigated so far. In this study, we reported the effects of an *in vivo* treatment on mammary glands with inactivated cultures of *Lactobacillus rhamnosus* of water buffaloes affected by subclinical mastitis in order to analyze the change in microbiota structure and evaluate the use of this GRAS as alternative strategy to the use of antibiotics. To the best of the knowledge of the authors, this was the first study using *Lactobacillus rhamnosu*s in an *in vivo* study on mammary gland. The scientific background behind the experimental design was that *Lactobacillus rhamnosus*, in combination with other Lactic acid bacteria, was able to modulate the pathogenic environment in the vaginal tract by regulating *Escherichia coli* infection and inflammation of the bovine endometrium [29]. At least so far, live cultures of probiotic were not found to improve mouse [46], cow [18,22,47] or ewe [24] mastitis. On the contrary, most of the Lactobacilli and Lactococci strains used so far for *in vivo* studies have been demonstrated to exert a pro-inflammatory activity: *Lactococcus lactis*, for example, is regarded as a pathogen causing mastitis [48,49]. The cultures used for *in vivo* challenging were previously inactivated with heat. This procedure was carried out to prevent any potential proinflammatory activity related to *in vivo* treatment with GRAS, as previously reported [50] which would have probably induced an acute inflammation, eventually switching the clinical status from sub-clinical to clinical mastitis. Moreover, a potential interference on the microbiota analysis of an uncontrolled overgrowth of living lactobacilli culture after inoculation in the mammary gland was envisaged, which would probably prevail over the other bacterial species, interfering with the detection of other microbial species.

We found that, although inactivated, the intramammary inoculation of LAB had a significant chemotactic effect toward leukocytes, as shown by the increase of milk somatic cells after 1 day from the inoculation of LAB. Intramammary gland treatment with PBS induces an increase of SCC as well, but two days after inoculation: interestingly, this result is consistent with what has been previously reported in a similar study using sterile PBS as negative control [51]. Treatment with antibiotics also elicited a chemotactic effect as well, although the increase of SCC is more limited as compared with LAB. The present results confirmed that intramammary inoculation of either bacteria, PBS or antibiotics triggers an inflammatory response, as demonstrated by the increase of SCC.

The microbiota of milk from affected animals largely corresponds to what has been previously reported [41] with some exceptions; among the others, the relative abundance of *Psycrobacter* and *Pseudomonas*, which were at 8.79% and 14.45% in the previous study, ranged in the present study from 2% to 4% and from 1% to 2%, respectively. S*B53* was not found as well, whereas its RA was at 3.7% in previous reports. These differences may be explained by the fact that sub-clinical mastitis can be caused by intramammary infection by a heterogeneous group of microorganisms, and the relative abundance of each microbial population may therefore be heterogeneous as well. No families nor genera were shared among subclinical mastitis samples, confirming that microbiota varies more in sub-clinical mastitis than healthy individuals as previously reported [41,52].

No major changes in microbiological cultures were found in milk quarters treated with LAB and PBS. As expected, the milk from quarters treated with antibiotic became negative at microbiological count, with one exception.

After treatment with inactivated Lactobacillus *rhamnosus*, we found an increase of up to 4% in the relative abundance of *Pseudomonas*. This finding is interesting, because the relative abundance of *Pseudomonas* genus was found to be associated to mastitis in water buffalo in our previous report [41], and is already known as mastitis pathogen in cow [53], sheep [54] and goats [55]. We may therefore hypothesize that the inoculation of LAB, though inactivated, may unbalance the microbiota of water buffalo by increasing the relative abundance of genus involved in the development of mastitis. It must also be said that the effects on microbiota of sterile PBS was more evident than the effects of inactivated cultures of LAB. This results may provide suggestions about the use of PBS as negative control for *in vivo* studies on mammary gland.

Other major finding was that the treatment with antibiotics increased at T6 the relative abundance of *Methylobacterium*, which was not found in milk treated with *Lactobacillus rhamnosus* and PBS. *Methylobacterium* forms biofilms and can develop resistance to high temperatures, drying, and disinfecting agents [56], which features may partially explain the growth capability of this genus after antibiotic treatment. These results supported in water buffalo the hypothesis that has been recently advanced in dairy cow that the mammary gland hosts a resilient microbiome that can reestablish after treatment with antibiotics [57].

Given the background that *Lactobacillus rhamnosus* culture was inactivated, and it induced an extravasation of leukocytes from blood toward the milk, we may speculate that the few modification of microbiota are determined by the intervening WBC, that are activated by the PAMP exposed on the surface of killed bacteria.

Interestingly, we found that no paracrine effect was present within the mammary glands: in all animals with subclincal mastitis quarters treated with antibiotic and other subclinical mastitis quarters treated with *Lactobacillus rhamnosus* or PBS, only the antibiotic-treated quarter became MC negative. About the others within the same mammary gland, they did not change or became MC positive at T6, suggesting the independence of every single quarter.

## Conclusions

This is the first experiment on water buffaloes, and in ruminants in general, that aimed to investigate the effect of *Lactobacillus rhamnosus* on subclinical mastitis. We demonstrated that the *in vivo* intramammary treatment with *Lactobacillus rhamnosus* has a transient pro-inflammatory activity as assessed by the SCC and is capable to modify the microbiota of milk after six days from inoculation, albeit slightly, even when the bacterial cultures were heat inactivated. This study confirmed the potential pro-inflammatory activity of GRAS bacteria, and suggests that careful approaches are needed for its *in vivo* use.

## Supporting information

S1 Table Relative abundance (> 1%) of microbiota taxa at family level. PBS: quarters treated with sterile PBS only, LAB: quarters treated with inactivated culture of *Lactobacillus rhamnosus* only, AB: quarters treated with antibiotics, as described in Material and Methods. T0: time zero; T6: time at 6 days post treatment.
(DOCX)

S2 Table Sampling time, microbiological result, SCC and group for each quarter included in the study. MC: Microbiological count. SCC (Somatic Cell Count) is x 1000. NA: not Assessed.
(DOCX)

S1 Fig Water buffalo milk taxonomic profile at family level (relative abundance of > 1%). PBS: quarters treated with sterile PBS only, LAB: quarters treated with inactivated culture of *Lactobacillus rhamnosus* only, Ab: quarters treated with antibiotics, as described in Material and Methods. T0: time zero; T6: time at 6 days post treatment.
(TIF)

## References

[1] Fao. FAO report, http://www.fao.org/agriculture/dairy-gateway/milk-production/dairy-animals/water-buffaloes/en/#.V-8-c_REfK8. 01/10/2016. 2016.

[2] Şahin A, Yıldırım A, Ulutaş Z. Changes in some physico-chemical content of Anatolian Buffalo milk according to the some environmental factors. Buffalo Bulletin. 2016;35: 573–585.

[3] Şahin A, Yıldırım A, Ulutaş Z. Effect of Various Environmental Factors and Management Practices on Somatic Cell Count in the Raw Milk of Anatolian Buffaloese. Pakistan Journal of Zoology. 2016;48: 325–332.

[4] Şahin A, Yıldırım A, Ulutaş Z. The effects of stage of lactation, parity and calving season on somatic cell counts in Anatolian Water Buffaloes. Indian Journal of Animal Science. 2017;51: 35–39.

[5] Thomas CS, Svennersten-Sjaunja K, Bhosrekar MR, Bruckmaier RM. Mammary cisternal size, cisternal milk and milk ejection in Murrah buffaloes. The Journal of dairy research. 2004;71: 162–8. Available: http://www.ncbi.nlm.nih.gov/pubmed/15190943 15190943 10.1017/s0022029904000081

[6] Moroni P, Sgoifo Rossi C, Pisoni G, Bronzo V, Castiglioni B, Boettcher PJ. Relationships between somatic cell count and intramammary infection in buffaloes. Journal of dairy science. 2006;89: 998–1003. 10.3168/jds.S0022-0302(06)72165-8 16507694

[7] Preethirani PL, Isloor S, Sundareshan S, Nuthanalakshmi V, Deepthikiran K, Sinha AY, et al Isolation, Biochemical and Molecular Identification, and In-Vitro Antimicrobial Resistance Patterns of Bacteria Isolated from Bubaline Subclinical Mastitis in South India. Kaltenboeck B, editor. PLOS ONE. 2015;10: e0142717 10.1371/journal.pone.0142717 26588070 PMC4654528

[8] Hogeveen H, Van Der Voort M. Assessing the economic impact of an endemic disease: the case of mastitis. Rev Sci Tech. 2017;36: 217–226. 10.20506/rst.36.1.2623 28926014

[9] Fagiolo A, Lai O. Mastitis in buffalo. Italian Journal of Animal Science. 2007;6: 200–206. 10.4081/ijas.2007.s2.200

[10] Halasa T, Huijps K, Østerås O, Hogeveen H. Economic effects of bovine mastitis and mastitis management: A review. Veterinary Quarterly. 2007;29: 18–31. 10.1080/01652176.2007.9695224 17471788

[11] Andersson DI, Hughes D. Persistence of antibiotic resistance in bacterial populations. FEMS Microbiology Reviews. 2011;35: 901–911. 10.1111/j.1574-6976.2011.00289.x 21707669

[12] Oliver S, Murinda S. Antimicrobial resistance of mastitis pathogens. Vet Clin North Am Food Anim Pract. 2012;28: 165–85. 10.1016/j.cvfa.2012.03.005 22664201

[13] Berghash SR, Davidson JN, Armstrong JC, Dunny GM. Effects of antibiotic treatment of nonlactating dairy cows on antibiotic resistance patterns of bovine mastitis pathogens. Antimicrobial Agents and Chemotherapy. 1983;24: 771–776. 10.1128/AAC.24.5.771 6660851 PMC185940

[14] Yang F, Wang Q, Wang X rong, Wang L, Li X pu, Luo J yin, et al Genetic characterization of antimicrobial resistance in Staphylococcus aureus isolated from bovine mastitis cases in Northwest China. Journal of Integrative Agriculture. Chinese Academy of Agricultural Sciences; 2016;15: 111111 10.1016/S2095-3119(16)61368-0

[15] Sharma C, Rokana N, Chandra M, Singh BP, Gulhane RD, Gill JPS, et al Antimicrobial Resistance: Its Surveillance, Impact, and Alternative Management Strategies in Dairy Animals. Frontiers in veterinary science. 2017;4: 237 10.3389/fvets.2017.00237 29359135 PMC5766636

[16] Gomes F, Henriques M. Control of Bovine Mastitis: Old and Recent Therapeutic Approaches. Current Microbiology. Springer US; 2016;72: 377–382. 10.1007/s00284-015-0958-8 26687332

[17] Malvisi M, Stuknytė M, Magro G, Minozzi G, Giardini A, De Noni I, et al Antibacterial activity and immunomodulatory effects on a bovine mammary epithelial cell line exerted by nisin A-producing Lactococcus lactis strains. Journal of Dairy Science. 2016;99: 2288–2296. 10.3168/jds.2015-10161 26774727

[18] Frola ID, Pellegrino MS, Espeche MC, Giraudo JA, Nader-Macias MEF, Bogni CI. Effects of intramammary inoculation of Lactobacillus perolens CRL1724 in lactating cows’ udders. Journal of Dairy Research. 2012;79: 84–92. 10.1017/S0022029911000835 22077995

[19] Bouchard DS, Rault L, Berkova N, Le Loir Y, Even S. Inhibition of Staphylococcus aureus invasion into bovine mammary epithelial cells by contact with live Lactobacillus casei. Applied and Environmental Microbiology. 2013;79: 877–885. 10.1128/AEM.03323-12 23183972 PMC3568545

[20] Bouchard DS, Seridan B, Saraoui T, Rault L, Germon P, Gonzalez-Moreno C, et al Lactic Acid Bacteria Isolated from Bovine Mammary Microbiota: Potential Allies against Bovine Mastitis. PloS one. 2015;10: e0144831 10.1371/journal.pone.0144831 26713450 PMC4694705

[21] Wu Q, Liu M, Yang J, Wang J, Zhu Y. Inflammation and Cell Damage via Attenuation of ASC-Independent NLRP3 Inflammasome Activation. 2016;82: 1173–1182. 10.1128/AEM.03044-15.Editor PMC4751844 26655757

[22] Crispie F, Alonso-Gómez M, O’Loughlin C, Klostermann K, Flynn J, Arkins S, et al Intramammary infusion of a live culture for treatment of bovine mastitis: Effect of live lactococci on the mammary immune response. Journal of Dairy Research. 2008;75: 374–384. 10.1017/S0022029908003385 18680623

[23] Klostermann K, Crispie F, Flynn J, Ross RP, Hill C, Meaney W. Intramammary infusion of a live culture of Lactococcus lactis for treatment of bovine mastitis: Comparison with antibiotic treatment in field trials. Journal of Dairy Research. 2008;75: 365–373. 10.1017/S0022029908003373 18680622

[24] Mignacca SA, Dore S, Spuria L, Zanghì P, Amato B, Duprè I, et al Intramammary infusion of a live culture of Lactococcus lactis in ewes to treat staphylococcal mastitis. Journal of Medical Microbiology. 2017;66: 1798–1810. 10.1099/jmm.0.000641 29134942

[25] Greene W, Gano A, Smith K, Hogan J, Todhunter D. Comparison of probiotic and antibiotic intramammary therapy of cattle with elevated somatic cell counts. J Dairy Sci. 1991;74: 2976–2981. 10.3168/jds.S0022-0302(91)78483-X 1779053

[26] Frola ID, Pellegrino MS, Magnano G, Giraudo JA, Espeche MC, Nader-Macias MEF, et al Histological examination of non-lactating bovine udders inoculated with Lactobacillus perolens CRL 1724. The Journal of dairy research. 2013;80: 28–35. 10.1017/S0022029912000581 23199568

[27] Marianelli C, Cifani N, Pasquali P. Evaluation of antimicrobial activity of probiotic bacteria against Salmonella enterica subsp. enterica serovar typhimurium 1344 in a common medium under different environmental conditions. Research in microbiology. 2010;161: 673–80. 10.1016/j.resmic.2010.06.007 20600855

[28] Johnson-Henry KC, Donato KA, Shen-Tu G, Gordanpour M, Sherman PM. Lactobacillus rhamnosus strain GG prevents enterohemorrhagic Escherichia coli O157:H7-induced changes in epithelial barrier function. Infection and immunity. 2008;76: 1340–8. 10.1128/IAI.00778-07 18227169 PMC2292865

[29] Genís S, Sánchez-Chardi A, Bach À, Fàbregas F, Arís A. A combination of lactic acid bacteria regulates Escherichia coli infection and inflammation of the bovine endometrium. Journal of Dairy Science. 2017;100: 479–492. 10.3168/jds.2016-11671 27837977

[30] Genís S, Bach À, Arís A. Effects of intravaginal lactic acid bacteria on bovine endometrium: Implications in uterine health. Veterinary Microbiology. 2017;204: 174–179. 10.1016/j.vetmic.2017.04.025 28532798

[31] Oh NS, Joung JY, Lee JY, Kim Y, Kim SH. Enhancement of Antioxidative and Intestinal Anti-inflammatory Activities of Glycated Milk Casein after Fermentation with Lactobacillus rhamnosus 4B15. Journal of agricultural and food chemistry. 2017;65: 4744–4754. 10.1021/acs.jafc.7b01339 28510450

[32] Amat S, Subramanian S, Timsit E, Alexander TW. Probiotic bacteria inhibit the bovine respiratory pathogen Mannheimia haemolytica serotype 1 in vitro. Letters in applied microbiology. 2017;64: 343–349. 10.1111/lam.12723 28178767

[33] Wu Q, Liu M-C, Yang J, Wang J-F, Zhu Y-H. Lactobacillus rhamnosus GR-1 Ameliorates Escherichia coli-Induced Inflammation and Cell Damage via Attenuation of ASC-Independent NLRP3 Inflammasome Activation. Applied and environmental microbiology. 2016;82: 1173–1182. 10.1128/AEM.03044-15 26655757 PMC4751844

[34] Wu Q, Zhu Y-H, Xu J, Liu X, Duan C, Wang M-J, et al Lactobacillus rhamnosus GR-1 Ameliorates Escherichia coli-Induced Activation of NLRP3 and NLRC4 Inflammasomes With Differential Requirement for ASC. Frontiers in microbiology. 2018;9: 1661 10.3389/fmicb.2018.01661 30087667 PMC6066506

[35] Addis MF, Tanca A, Uzzau S, Oikonomou G, Bicalho RC, Moroni P. The bovine milk microbiota: insights and perspectives from -omics studies. Molecular bioSystems. 2016;12: 2359–72. 10.1039/c6mb00217j 27216801

[36] Bicalho RC arvalho. Microbiota of cow’s milk; distinguishing healthy, sub-clinically and clinically diseased quarters. PloS one. 2014;9: e85904 10.1371/journal.pone.0085904 24465777 PMC3896433

[37] Lima SF, Bicalho ML de S, Bicalho RC. Evaluation of milk sample fractions for characterization of milk microbiota from healthy and clinical mastitis cows. Plos One. 2018;13: e0193671 10.1371/journal.pone.0193671 29561873 PMC5862444

[38] Oikonomou G, Machado VS, Santisteban C, Schukken YH, Bicalho RC. Microbial Diversity of Bovine Mastitic Milk as Described by Pyrosequencing of Metagenomic 16s rDNA. PLoS ONE. 2012;7 10.1371/journal.pone.0047671 23082192 PMC3474744

[39] Kuehn JS, Gorden PJ, Munro D, Rong R, Dong Q, Plummer PJ, et al Bacterial Community Profiling of Milk Samples as a Means to Understand Culture-Negative Bovine Clinical Mastitis. PLoS ONE. 2013;8 10.1371/journal.pone.0061959 23634219 PMC3636265

[40] Vayssier-Taussat M, Albina E, Citti C, Cosson J-F, Jacques M-A, Lebrun M-H, et al Shifting the paradigm from pathogens to pathobiome: new concepts in the light of meta-omics. Frontiers in cellular and infection microbiology. 2014;4: 29 10.3389/fcimb.2014.00029 24634890 PMC3942874

[41] Catozzi C, Sanchez Bonastre A, Francino O, Lecchi C, De Carlo E, Vecchio D, et al The microbiota of water buffalo milk during mastitis. PLoS ONE. 2017;12: 1–20. 10.1371/journal.pone.0184710 28926595 PMC5604978

[42] Caporaso JG, Kuczynski J, Stombaugh J, Bittinger K, Bushman FD, Costello EK, et al NIH Public Access. 2011;7: 335–336. 10.1038/nmeth.f.303.QIIME

[43] Callahan BJ, Mcmurdie PJ, Rosen MJ, Han AW, A AJ. HHS Public Access. 2016;13: 581–583. 10.1038/nmeth.3869.DADA2 PMC4927377 27214047

[44] DeSantis TZ, Hugenholtz P, Larsen N, Rojas M, Brodie EL, Keller K, et al Greengenes, a chimera-checked 16S rRNA gene database and workbench compatible with ARB. Applied and Environmental Microbiology. 2006;72: 5069–5072. 10.1128/AEM.03006-05 16820507 PMC1489311

[45] Bokulich NA, Zhang Y, Dillon M, Rideout JR, Bolyen E, Li H, et al q2-longitudinal: a QIIME 2 plugin for longitudinal and paired-sample analyses of microbiome data. biorXIV. 2017; 10.1101/223974 PMC6247016 30505944

[46] Camperio C, Armas F, Biasibetti E, Frassanito P, Giovannelli C, Spuria L, et al A mouse mastitis model to study the effects of the intramammary infusion of a food-grade Lactococcus lactis strain. PloS one. 2017;12: e0184218 10.1371/journal.pone.0184218 28873396 PMC5584933

[47] Beecher C, Daly M, Berry DP, Klostermann K, Flynn J, Meaney W, et al Administration of a live culture of Lactococcus lactis DPC 3147 into the bovine mammary gland stimulates the local host immune response, particularly IL-1β and IL-8 gene expression. Journal of Dairy Research. 2009;76: 340 10.1017/S0022029909004154 19445831

[48] Plumed-Ferrer C, Uusikylä K, Korhonen J, von Wright A. Characterization of Lactococcus lactis isolates from bovine mastitis. Veterinary microbiology. 2013;167: 592–9. 10.1016/j.vetmic.2013.09.011 24080351

[49] Werner B, Moroni P, Gioia G, Lavín-Alconero L, Yousaf A, Charter ME, et al Short communication: Genotypic and phenotypic identification of environmental streptococci and association of Lactococcus lactis ssp. lactis with intramammary infections among different dairy farms. Journal of dairy science. 2014;97: 6964–9. 10.3168/jds.2014-8314 25242419

[50] Rainard P. Mammary microbiota of dairy ruminants: fact or fiction? Veterinary research. 2017;48: 25 10.1186/s13567-017-0429-2 28412972 PMC5392980

[51] Tassi R, McNeilly TN, Fitzpatrick JL, Fontaine MC, Reddick D, Ramage C, et al Strain-specific pathogenicity of putative host-adapted and nonadapted strains of Streptococcus uberis in dairy cattle. Journal of dairy science. 2013;96: 5129–45. 10.3168/jds.2013-6741 23769372

[52] Zaneveld JR, McMinds R, Thurber RV. Stress and stability: Applying the Anna Karenina principle to animal microbiomes. Nature Microbiology. 2017;2 10.1038/nmicrobiol.2017.121 28836573

[53] Hertl JA, Schukken YH, Bar D, Bennett GJ, González RN, Rauch BJ, et al The effect of recurrent episodes of clinical mastitis caused by gram-positive and gram-negative bacteria and other organisms on mortality and culling in Holstein dairy cows. Journal of Dairy Science. Elsevier; 2011;94: 4863–4877. 10.3168/jds.2010-4000 21943738

[54] Wright EA, Di Lorenzo V, Trappetti C, Liciardi M, Orru G, Viti C, et al Divergence of a strain of Pseudomonas aeruginosa during an outbreak of ovine mastitis. Veterinary Microbiology. Elsevier B.V.; 2015;175: 105–113. 10.1016/j.vetmic.2014.11.011 25475851

[55] Scaccabarozzi L, Leoni L, Ballarini A, Barberio A, Locatelli C, Casula A, et al Pseudomonas aeruginosa in dairy goats: Genotypic and phenotypic comparison of intramammary and environmental isolates. PLoS ONE. 2015;10 10.1371/journal.pone.0142973 26606430 PMC4659641

[56] Kovaleva J, Degener JE, van der Mei HC. Methylobacterium and its role in health care-associated infection. Journal of clinical microbiology. 2014;52: 1317–21. 10.1128/JCM.03561-13 24430456 PMC3993692

[57] Ganda EK, Gaeta N, Sipka A, Pomeroy B, Oikonomou G, Schukken YH, et al Normal milk microbiome is reestablished following experimental infection with Escherichia coli independent of intramammary antibiotic treatment with a third-generation cephalosporin in bovines. Microbiome. 2017;5: 74 10.1186/s40168-017-0291-5 28701174 PMC5506599

