# Supplementary material for: Impact of intramammary inoculation of inactivated Lactobacillus rhamnosus and antibiotics on the milk microbiota of water buffalo with subclinical mastitis
Source: PLoS One. 2019 Jan 7;14(1):e0210204. doi: 10.1371/journal.pone.0210204 (PMC6322744; doi:10.1371/journal.pone.0210204)
Supplement: S1 Table — PBS: quarters treated with sterile PBS only, LAB: quarters treated with inactivated culture of Lactobacillus rhamnosus only, AB: quarters treated with antibiotics, as described in Material and Methods. T0: time zero; T6: time at 6 days post treatment. (DOCX) [file pone.0210204.s001.docx]

**S1 Table. Relative abundance (> 1%) of microbiota taxa at family level**

**PBS: quarters treated with sterile PBS only, LAB: quarters treated with inactivated culture of *Lactobacillus rhamnosus* only, AB: quarters treated with antibiotics, as described in Material and Methods. T0: time zero; T6: time at 6 days post treatment.**

|  | ***Relative abundance frequencies*** | | | | | | ***p-value (where p < 0.05)*** | | | | | |
| --- | --- | --- | --- | --- | --- | --- | --- | --- | --- | --- | --- | --- |
|  | **Quarter treated with PBS** | | **Quarters treated with LAB** | | **Quarters treated with antibiotics** | | **PBS** | **LAB** | **Ab** | **T5** | **T5** | **T5** |
|  | **SM_PBS_T0** | **SM_PBS_T5** | **SM_L_T0** | **SM_L_T5** | **SM_A_T0** | **SM_A_T5** | **T0 vs T5** | **T0 vs T5** | **T0 vs T5** | **PBS vs LAB** | **PBS vs AB** | **LAB vs AB** |
| Actinomycetaceae | 0,78% | 0,3% | 0,3% | 0,3% | 0,3% | 0,5% | ns | ns | ns | ns | ns | ns |
| Corynebacteriaceae | 3,46% | 4,8% | 3,3% | 3,7% | 6,4% | 7,4% | ns | ns | ns | ns | ns | 0.04 |
| Dietziaceae | 0,42% | 0,7% | 0,5% | 0,5% | 1,0% | 1,3% | ns | ns | ns | ns | ns | ns |
| Microbacteriaceae | 0,07% | 0,4% | 0,2% | 0,3% | 0,2% | 2,5% | ns | ns | 0.04 | ns | 0.02 | ns |
| Micrococcaceae | 1,80% | 2,0% | 1,1% | 1,0% | 0,9% | 3,0% | ns | ns | ns | ns | ns | 0.02 |
| Propionibacteriaceae | 2,61% | 2,4% | 1,8% | 1,8% | 2,8% | 9,8% | ns | ns | ns | ns | ns | 0.002 |
| Bacteroidaceae | 0,59% | 1,5% | 0,8% | 1,2% | 1,2% | 0,5% | ns | ns | ns | ns | ns | ns |
| Cytophagaceae | 0,56% | 0,5% | 0,0% | 0,1% | 0,1% | 6,1% | ns | ns | ns | ns | 0.02 | 0.04 |
| [Weeksellaceae] | 0,23% | 0,4% | 0,2% | 0,2% | 0,1% | 0,9% | ns | ns | ns | ns | ns | ns |
| Flavobacteriaceae | 0,39% | 0,8% | 0,7% | 1,1% | 0,4% | 1,8% | ns | ns | ns | ns | ns | ns |
| Bacillaceae | 0,63% | 0,7% | 0,7% | 0,8% | 1,3% | 0,9% | ns | ns | ns | ns | ns | ns |
| Planococcaceae | 0,59% | 3,7% | 1,1% | 1,0% | 0,7% | 0,9% | ns | ns | ns | ns | ns | ns |
| Staphylococcaceae | 34,25% | 26,9% | 44,3% | 45,9% | 42,5% | 7,5% | ns | ns | ns | ns | ns | 0.02 |
| Aerococcaceae | 5,19% | 4,2% | 4,9% | 3,4% | 5,9% | 6,6% | ns | ns | ns | ns | ns | ns |
| Streptococcaceae | 5,50% | 6,2% | 10,8% | 8,3% | 1,2% | 1,6% | ns | ns | ns | ns | ns | ns |
| [Tissierellaceae] | 0,29% | 0,4% | 0,1% | 0,3% | 0,7% | 0,7% | ns | ns | ns | ns | ns | ns |
| Clostridiaceae | 0,46% | 0,5% | 0,2% | 0,3% | 0,8% | 1,0% | ns | ns | ns | ns | ns | ns |
| Lachnospiraceae | 1,00% | 0,6% | 1,2% | 0,9% | 1,1% | 0,6% | ns | ns | ns | ns | ns | ns |
| Peptostreptococcaceae | 0,69% | 0,9% | 1,0% | 0,4% | 0,6% | 0,8% | ns | ns | ns | 0,04 | ns | 0.04 |
| Ruminococcaceae | 2,65% | 2,5% | 2,3% | 2,9% | 4,7% | 2,1% | ns | ns | ns | ns | ns | ns |
| Bradyrhizobiaceae | 1,35% | 1,4% | 1,3% | 0,9% | 0,2% | 1,4% | ns | ns | ns | ns | ns | ns |
| Methylobacteriaceae | 1,34% | 5,2% | 1,0% | 1,8% | 1,1% | 6,7% | ns | ns | 0,03 | ns | ns | ns |
| Xanthobacteraceae | 0,00% | 0,1% | 0,0% | 0,0% | 0,0% | 0,0% | ns | ns | ns | ns | ns | ns |
| Rhodobacteraceae | 0,10% | 0,6% | 0,2% | 0,4% | 1,6% | 0,7% | 0,03 | ns | ns | ns | ns | ns |
| Sphingomonadaceae | 0,43% | 1,3% | 0,4% | 0,8% | 0,7% | 2,5% | ns | ns | ns | ns | ns | ns |
| Comamonadaceae | 1,67% | 2,2% | 1,4% | 0,4% | 1,4% | 2,5% | ns | ns | ns | 0,04 | ns | 0,008 |
| Rhodocyclaceae | 0,98% | 1,0% | 0,4% | 0,6% | 0,1% | 1,2% | ns | ns | 0.02 | ns | ns | ns |
| Enterobacteriaceae | 5,42% | 0,8% | 2,4% | 0,5% | 1,8% | 3,8% | 0.03 | ns | ns | ns | ns | ns |
| Halomonadaceae | 0,93% | 1,5% | 0,5% | 1,8% | 0,6% | 2,4% | ns | ns | ns | ns | ns | ns |
| Moraxellaceae | 10,36% | 6,5% | 2,8% | 2,3% | 2,5% | 2,8% | ns | ns | ns | ns | ns | ns |
| Pseudomonadaceae | 1,93% | 3,5% | 1,5% | 5,1% | 1,7% | 3,5% | ns | 0.006 | ns | ns | ns | ns |
| Xanthomonadaceae | 0,46% | 0,4% | 0,1% | 0,5% | 0,5% | 1,0% | ns | ns | ns | ns | ns | ns |
| Other | 12,84% | 15,1% | 12,4% | 10,5% | 14,8% | 14,8% | ns | ns | ns | ns | ns | ns |
