# Supplementary material for: Impact of intramammary inoculation of inactivated Lactobacillus rhamnosus and antibiotics on the milk microbiota of water buffalo with subclinical mastitis
Source: PLoS One. 2019 Jan 7;14(1):e0210204. doi: 10.1371/journal.pone.0210204 (PMC6322744; doi:10.1371/journal.pone.0210204)
Supplement: S2 Table — MC: Microbiological count. SCC (Somatic Cell Count) is x 1000. NA: not Assessed. (DOCX) [file pone.0210204.s002.docx]

**S2 Table. Sampling time, microbiological result, SCC and group for each quarter included in the study**

**MC: Microbiological count. SCC (Somatic Cell Count) is x 1000. NA: not Assessed**

|  |  | **T-28** | | **T-21** | | **T-15** | | **T-7** | | **T0** | | **T1** | | **T2** | | **T6** | |
| --- | --- | --- | --- | --- | --- | --- | --- | --- | --- | --- | --- | --- | --- | --- | --- | --- | --- |
| **ID** | **Treatment** | **MC** | **SCC** | **MC** | **SCC** | **MC** | **SCC** | **MC** | **SCC** | **MC** | **SCC** | **MC** | **SCC** | **MC** | **SCC** | **MC** | **SCC** |
| 4 | LAB | Group B Strep/ Bacilli | 1866,5 | S. aureus | 1068 | S. aureus / Group B Strep | 2968 | S.agalactiae | 2221 | S. agalactiae | 373 | Negative | ND | CNS | 22458 | CNS/  S.agalactiae | 559 |
| 5 | LAB | S.aureus | 198,5 | Negative | 80 | S. aureus | 133 | S. aureus | 136 | S. aureus | 148 | S.aureus | 9044 | S.aureus | 3475 | S.aureus | 703 |
| 6 | LAB | Bacilli | 102 | Negative | 39 | S. aureus | 21 | S. aureus | 16 | CNS | 566 | S.aureus | 13489 | S.aureus | 12987 | S.aureus/  S.agalactiae | 682 |
| 11 | LAB | S.aureus | 156 | S. aureus/Group B Strep | 88 | S. aureus | 992 | S. aureus | 86 | S. agalactiae | 19407 | S.aureus | 5328 | S.aureus | 1210 | S.aureus | 450 |
| 30 | LAB | S.aureus | 428 | S. aureus | 151 | S. aureus | 306 | S. aureus | 473 | S. aureus | 34 | Negative | 14895 | Negative | 1969 | S.aureus | 356 |
| 32 | LAB | S.aureus | 681 | S. aureus e Bacilli | 567 | S. aureus | 533 | CNS | 268 | CNS | 27 | Negative | 6705 | CNS | 5257 | S.aureus | 669 |
| 36 | LAB | S.aureus/ Group B Strep / E. coli | 149 | Group B Strep/ Enterococcus | 430 | Group B Strep | 864 | Negative | 309 | S. aureus | 111 | Negative | 18646 | Negative | 8598 | CNS | 152 |
| 44 | LAB | S.aureus | 1298 | S. aureus | 2042 | S. aureus | 827 | S. aureus | 879 | S. aureus | 232 | Negative | 19986 | S.aureus | 7567 | S.aureus/  S.agalactiae | 179 |
| 57 | LAB | S.aureus/  E. coli | 28 | CNS | 46 | CNS | 13 | CNS | 36 | CNS | 147 | CNS | 7891 | Negative | 1114 | S.aureus | 1533 |
| 74 | LAB | S.aureus | 415 | S. aureus | 2489 | S. aureus | 1851 | S. aureus | 831 | S. aureus | 3210 | S.aureus | 9424 | Negative | 2213 | S.aureus | 3374 |
| 75 | LAB | S.aureus/  E. coli /  Bacilli | 591 | S. aureus | 333 | S. aureus | 1413 | S. aureus | 494 | S. aureus | 3130 | S.aureus | 3165 | S.aureus | 1098 | CNS/  S.agalactiae | 110 |
| 80 | LAB | S.aureus | 182,5 | S. aureus | 265 | S. aureus | 310 | S. aureus | 1436 | S. aureus | 439 | Negative | 11160 | S.aureus | 3910 | CNS | 1214 |
| 79 | LAB | NA | NA | S. aureus | 96 | S. aureus | 32 | Negative | ND | S. aureus | 456 | Negative | 17323 | S.aureus | 1520 | S.aureus | 747 |
| 12 | LAB | S.aureus | 503,5 | S. aureus /Group B Strep | 82 | Group B Strep/ S. aureus | 1644 | S. aureus/  S.agalactiae | 131 | S. aureus | ND | S.aureus/  S.agalactiae | 2486 | S.aureus | 1122 | S.aureus | 284 |
| 68 | LAB | S.aureus | 5187 | Group B Strep | 402 | Group B Strep | 138 | S.agalactiae | 245 | S. agalactiae | 85 | Negative | 11124 | Negative | 2740 | S.aureus | 3484 |
| 13 | AB | S.aureus | 159 | S. aureus | 253 | S. aureus | 340 | S. aureus | 378 | S. aureus | 948 | Negative | 4577 | Negative | 9588 | S.aureus | 850 |
| 15 | AB | S.aureus / Bacilli | 23,5 | S. aureus /  Bacilli / Enterococcus | 18 | S. aureus | 18 | S. aureus | 28 | S. aureus | 20 | Negative | 462 | Negative | 4735 | Negativo | 514 |
| 31 | AB | S.aureus | 283 | Staph e Bacilli | 1037 | S. aureus | 230 | CNS | 698 | S. aureus | 913 | Negative | 1307 | Negative | 5257 | Negativo | 113 |
| 39 | AB | S.aureus / Bacilli | 950 | S. aureus | 202 | S. aureus | 765 | S. aureus | 1620 | S. aureus | 1271 | Negative | 3351 | Negative | 2834 | Negativo | 152 |
| 70 | AB | S.aureus | 2317 | S. aureus | 2195 | S. aureus | 879 | S. aureus | 3090 | S. aureus | 1733 | Negative | ND | Negative | ND | Negativo | 236 |
| 76 | AB | S.aureus /  E. coli | 4416,5 | S. aureus | 589 | S. aureus | 341 | S. aureus | 1973 | S. aureus | 102 | Negative | 3615 | Negative | 3747 | Negativo | 492 |
| 78 | AB | S.aureus | 199,5 | S. aureus | 135 | S. aureus | 435 | S. aureus | 195 | S. aureus | 4162 | Negative | 1978 | Negative | ND | Negativo | 215 |
| 22 | AB | S.aureus / Group B Strep | 40 | Bacilli | 229 | Negative | 37 | CNS | 19 | CNS | 329 | Negative | 198 | Negative | 1706 | Negativo | 263 |
| 67 | AB | S.aureus / Group B Strep / E. coli | 929,5 | Group B Strep | 343 | Group B Strep | 274 | S.agalactiae | 272 | S. aureus | 938 | Negative | 2340 | Negative | 3626 | Negativo | 214 |
| 71 | AB | S.aureus / E. coli | 1456 | S. aureus /  Bacilli /  Group B Strep | 18196 | S. aureus | 1510 | S. aureus | ND | CNS | 36 | Negative | 6860 | Negative | 7155 | Negativo | 478 |
| 73 | AB | Negative | 26370 | S. aureus | 155 | S. aureus | 418 | S.aureus | 661 | CNS/  S.agalactiae | 224 | Negative | ND | Negative | 2859 | Negativo | ND |
| 7 | PBS | S.aureus / Group B Strep | 3462,5 | Negative | 2494 | S. aureus / Group B Strep | 1052 | S.aureus/  S.agalactiae | 1753 | S.aureus/  S.agalactiae | 397 | S.aureus/  S.agalactiae | 1300 | S.aureus/  S.agalactiae | 1546 | CNS | 120 |
| 8 | PBS | S.aureus | 1701 | Negative | 936 | S. aureus | 405 | S. aureus | 634 | CNS/  S.agalactiae | 543 | S.aureus | 3271 | S.aureus | 2122 | CNS | 427 |
| 16 | PBS | S.aureus | 163 | S. aureus | 511 | S. aureus | 402 | S. aureus | 394 | S. agalactiae | 3864 | Negative | 1132 | Negative | 747 | CNS | 1582 |
| 21 | PBS | S.aureus / E. coli | 372,5 | S. aureus | 978 | S. aureus | 894 | CNS | 1311 | S.aureus/  S.agalactiae | 3105 | CNS | 1650 | CNS | 1367 | CNS/  S.agalactiae | 4574 |
| 24 | PBS | S.aureus | 363 | S. aureus | 1862 | S. aureus | 547 | S. aureus | 1172 | S. aureus | ND | CNS | 3388 | CNS | 1411 | S.aureus/  S.agalactiae | 7320 |
| 41 | PBS | S.aureus | 1015,5 | S. aureus | 292 | S. aureus | 152 | S. aureus | 225 | S. aureus | 1110 | Negative | 401 | S.aureus | 352 | S.aureus | 527 |
| 49 | PBS | Bacilli | 47,5 | Negative | 23 | S. aureus | 15 | Negative | 27 | S. aureus | 616 | S.aureus | 15748 | Negative | 6562 | S.aureus | 249 |
| 54 | PBS | S.aureus / Group B Strep | 2847,5 | S. aureus /  Group B Strep | 2794 | Group B Strep / CNS | 2242 | CNS/S.agalactiae | 1500 | S. aureus | 27 | S.agalactiae | 749 | Negative | 3569 | S.aureus | ND |
| 55 | PBS | S.aureus / Group B Strep | 8241,5 | S. aureus /  Group B Strep | 8508 | Group B Strep | 12172 | Strepto | 7516 | S. aureus | 227 | S.agalactiae | 1349 | S.agalactiae | 17267 | S.aureus/  S.agalactiae | 594 |
| 56 | PBS | Bacilli | 8542,5 | S. aureus /  Group B Strep | 1695 | Group B Strep / S. aureus | 2797 | CNS / Strepto | 3880 | CNS | 854 | Negative | 1338 | Negative | 4497 | S.aureus | 240 |
| 58 | PBS | S.aureus /  E. coli | 232,5 | CNS | 718 | S. aureus | 175 | S. aureus | 180 | CNS | 809 | S.aureus | 369 | S.aureus | 353 | S.aureus | 546 |
| 66 | PBS | S.aureus | 1308,5 | S. aureus | 223 | S. aureus | 841 | S. aureus | 409 | S. aureus | ND | S.aureus | 3123 | S.aureus | 1266 | S.aureus | 310 |
| 69 | PBS | S.aureus /  E. coli | 1350 | S. aureus | 845 | S. aureus | 603 | S. aureus | 836 | CNS | 22 | S.aureus | 2240 | S.aureus | ND | Negativo | 611 |
| 43 | PBS | S.aureus | 37,5 | Negative | 51 | Negative | 51 | S. aureus | 93 | S. aureus | 758 | S.aureus | 97 | Negative | 668 | Negativo | 397 |
| 59 | PBS | S.aureus /  E. coli /  Bacilli | 18,5 | Negative | 24 | S. aureus | 13 | Negative | 32 | CNS | 22 | S.aureus | 18819 | S.aureus | 6900 | S.aureus/  S.agalactiae | 549 |
| 60 | PBS | S.aureus /  E. coli | 24 | Negative | 53 | S. aureus | 604 | S. aureus | 323 | S. aureus | 72 | S.aureus | 683 | S.aureus | 671 | S.aureus | 498 |
| 65 | PBS | E. coli | 50,5 | Negative | 17 | S. aureus | 26 | Negative | 40 | S. aureus | 196 | Enterococcus | 2290 | Negative | 15430 | Negativo | 230 |
