# Supplementary figures and images for: Impact of intramammary inoculation of inactivated Lactobacillus rhamnosus and antibiotics on the milk microbiota of water buffalo with subclinical mastitis
Source: PLoS One. 2019 Jan 7;14(1):e0210204. doi: 10.1371/journal.pone.0210204 (PMC6322744; doi:10.1371/journal.pone.0210204)

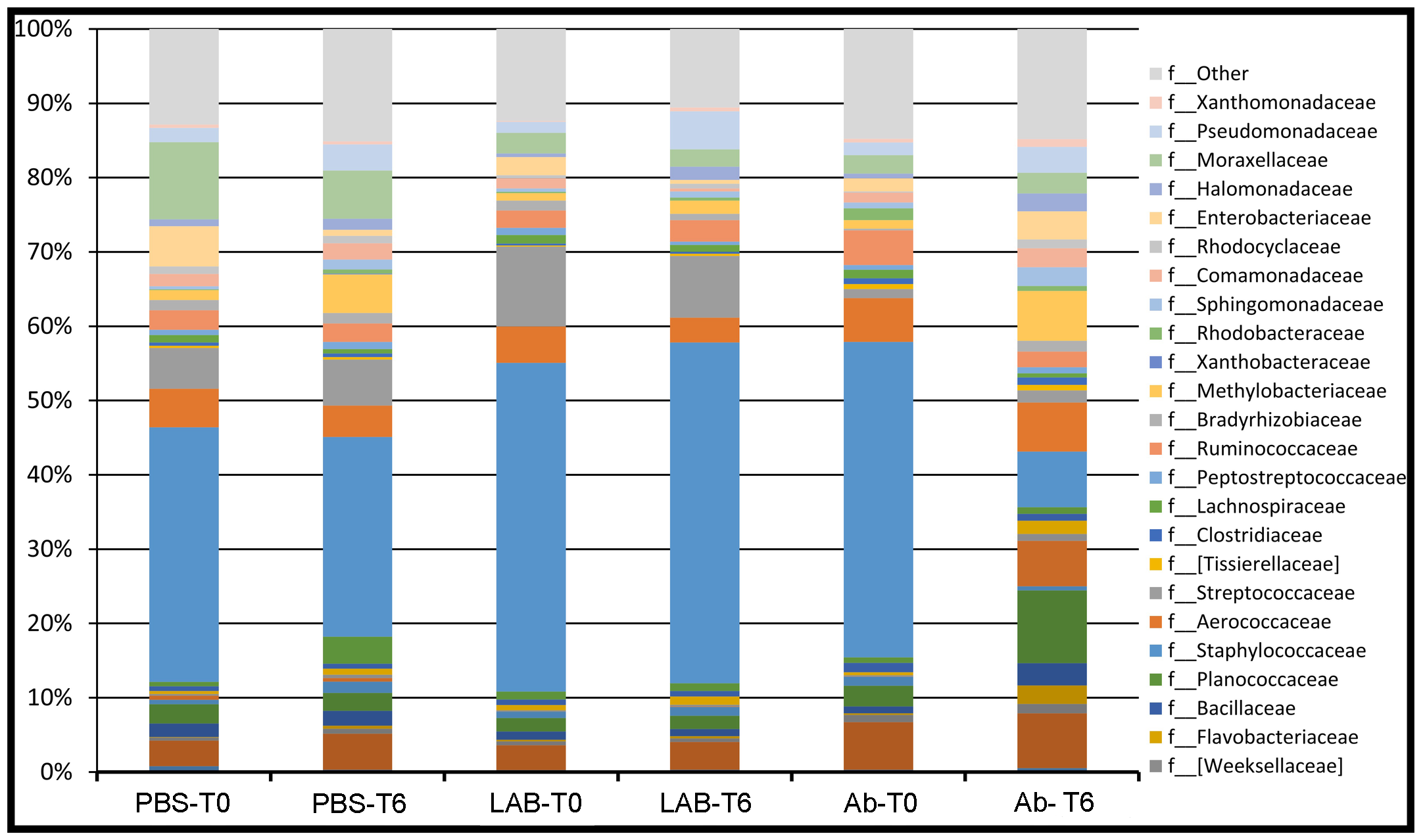

Supplement: S1 Fig — PBS: quarters treated with sterile PBS only, LAB: quarters treated with inactivated culture of Lactobacillus rhamnosus only, Ab: quarters treated with antibiotics, as described in Material and Methods. T0: time zero; T6: time at 6 days post treatment. (TIF) [file pone.0210204.s003.tif]
